# Drought Stress Stimulates the Terpenoid Backbone and Triterpenoid Biosynthesis Pathway to Promote the Synthesis of Saikosaponin in *Bupleurum chinense* DC. Roots

**DOI:** 10.3390/molecules27175470

**Published:** 2022-08-25

**Authors:** Linlin Yang, Lu Qiao, Xiuhong Su, Baoyu Ji, Chengming Dong

**Affiliations:** 1 Henan Provincial Ecological Planting Engineering Technology Research Center of Daodi Herbs, School of Pharmacy, Henan University of Chinese Medicine, Zhengzhou 450046, China; 2 Co-Construction Collaborative Innovation Centre for Chinese Medicine and Respiratory Diseases by Henan & Education Ministry of PR China, Henan University of Chinese Medicine, Zhengzhou 450046, China

**Keywords:** *Bupleurum chinense* DC., drought stress, terpenoid backbone and triterpenoid biosynthesis, plant hormones, saikosaponins

## Abstract

*Bupleurum chinense* is an important medicinal plant in China; however, little is known regarding how this plant transcribes and synthesizes saikosaponins under drought stress. Herein, we investigated how drought stress stimulates the transcriptional changes of *B. chinense* to synthesize saikosaponins. Short-term drought stress induced the accumulation of saikosaponins, especially from the first re-watering stage (RD_1 stage) to the second re-watering stage (RD_2 stage). Saikosaponin-a and saikosaponin-d increased by 84.60% and 75.13%, respectively, from the RD_1 stage to the RD_2 stage. Drought stress also stimulated a rapid increase in the levels of the hormones abscisic acid, salicylic acid, and jasmonic acid. We screened 49 Unigenes regarding the terpenoid backbone and triterpenoid biosynthesis, of which 33 differential genes were significantly up-regulated during drought stress. Moreover, one P450 and two UGTs are possibly involved in the synthesis of saikosaponins, while some transcription factors may be involved in regulating the expression of key enzyme genes. Our study provides a reference for the cultivation of *B. chinense* and a practical means to ensure the quality (safety and effectiveness) of *B. chinense* for medicinal use, as well as insights into the modernization of the China Agriculture Research System.

## 1. Introduction

Reasonable cultivation measures are the basis of producing high-quality Chinese herbal materials. The rationality of cultivation, the safety of herbal materials, and the effectiveness of curative effects are the realistic requirements of the China Agriculture Research System (CARS) for Chinese herbal products [1]. *Bupleurum chinense* DC. (*B. chinense*) is a plant in the family Umbelliferae. The Chinese Pharmacopoeia 2020 indicates that BUPLEURI RADIX, a traditional Chinese medicine [2], is based on *B. chinense*, amongst other plants [3]. Several prescriptions use BUPLEURI RADIX, including the Xiaochaihu decoction and the Qingfei Paidu decoction, which showed efficacy against Coronavirus Disease 2019 (COVID-19) [4]. The Xiaochaihu decoction is a traditional Chinese medicine composed of BUPLEURI RADIX, PINELLIAE RHIZOMA, GINSENG RADIX ET RHIZOMA, GLYCYRRHIZAE RADIX ET RHIZOMA, etc. It is clinically used to treat cold, influenza, chronic hepatitis, and other diseases. The Qingfei Paidu decoction is a traditional Chinese medicine compound for treating pneumonia caused by COVID-19, and consists of BUPLEURI RADIX and other Chinese herb compounds. Saikosaponins are the material basis for the efficacy of *B. chinense*. Saikosaponins are mainly distributed in the roots of *B. chinense*, while the content of saikosaponins in the aboveground parts, such as stems and leaves, is extremely low. However, the wide planting area and modest planting technology of *B. chinense* cultivation have affected its quality as medicinal material [5]. The total amount of saikosaponin-a (SS-a) and saikosaponin-d (SS-d) in 29 production areas in 11 provinces, cities, and autonomous regions in China ranges from 0.29 to 1.52%, which limits the efficacy of *B. chinense* [6]. Therefore, understanding the molecular mechanism underlying *B. chinense* formation and correspondingly developing the cultivation technology for *B. chinense* is important for realizing the stable and controllable quality of medicinal materials [7].

*Bupleurum chinense* DC. is widely distributed in arid and semi-arid areas of China, with a large geographical span [8]. Its quality in terms of the content of saikosaponins, its active ingredient, is mostly affected by the external environment [9,10]. The so-called intrinsic genotype determines the property of a traditional Chinese medicine, and is regulated by external ecological factors [7]. The biosynthesis of saikosaponin is a complex process affected by basic and secondary metabolism in *B. chinense* [11], and remains poorly understood. Saikosaponin biosynthesis includes three stages: (1) the glycolytic product acetyl-CoA is converted into the precursors 3-isoprene pyrophosphate (IPP) and dimethylallyl pyrophosphate (DMAPP) via the mevalonic acid pathway (MVA) [12]; (2) IPP and DMAPP are catalyzed into 2,3-oxidized squalene via various enzymes and intermediates [13]; (3) various monomer saikosaponins are synthesized via cyclization, hydroxylation, and glycosylation of 2,3-oxidized squalene [14,15]. However, the genes involved in saikosaponin biosynthesis are not fully characterized, and their regulation by external ecological factors remains unclear [16,17]. Therefore, understanding the secondary metabolic process of saikosaponin synthesis and the mechanism underlying its molecular regulation in terms of the intrinsic gene expression and external ecological factors (drought) remains essential.

RNA sequencing (RNA-seq) has been commonly used in transcriptomic research [18]. The new-generation sequencing technology provides biological transcription data more accurately than that previously used [19]. The formation of saikosaponin in *B. chinense* is the result of gene expression under the regulation of the external environment, especially drought [20]. Therefore, the in-depth mining of relevant genes regulating the secondary metabolism network of *B. chinense* using transcriptome sequencing is helpful to analyze the responses of transcripts to drought and the molecular mechanism regulating the synthesis and accumulation of saikosaponins using RNA-seq technology [21,22]. Sui et al. have conducted preliminary transcriptome sequencing of *B. chinense* [23,24], but data regarding the transcriptome changes of *B. chinense* under drought regulation are lacking.

In this study, we used RNA-seq technology for the first time—to the best of our knowledge—to analyze how drought stress stimulates the terpene main chain and the triterpene biosynthesis pathway in *B. chinense* to promote the synthesis of saikosaponins. In addition, ultra-performance liquid chromatography (UHPLC-ESI-MS/MS) was used to detect abscisic acid (ABA), jasmonic acid (JA), and salicylic acid (SA) in *B. chinense*, to analyze the relationship between the transcriptional information and changes in endogenous hormones during drought stress. The pattern of expression of the genes encoding key enzymes involved in saikosaponin synthesis was also analyzed using Real-time Quantitative PCR (qPCR). The results of the transcriptome sequencing and the changes in saikosaponin content were used to screen cytochrome P450 (CYP450) and glycosyltransferase (UGT) to assess their possible involvement in saikosaponin synthesis. We analyzed the transcription factors in five families, namely ethylene-responsive factor (ERF), basic/helix-loop-helix (bHLH), MYB transcription factor (MYB), WRKY transcription factor (WRKY), and NAC transcription factor (NAC). The transcription factors possibly involved in regulating gene expression were also screened. Finally, we preliminary constructed the regulatory network for the synthesis of saikosaponin under drought stress. Our results provide deeper insights into the molecular mechanism underlying saikosaponin formation in *B. chinense* under drought stress, which is significant for improving the water supply and the overall technology and process for cultivating *B. chinense.*

## 2. Results

### 2.1. Saikosaponin Content

**Figure 1:**
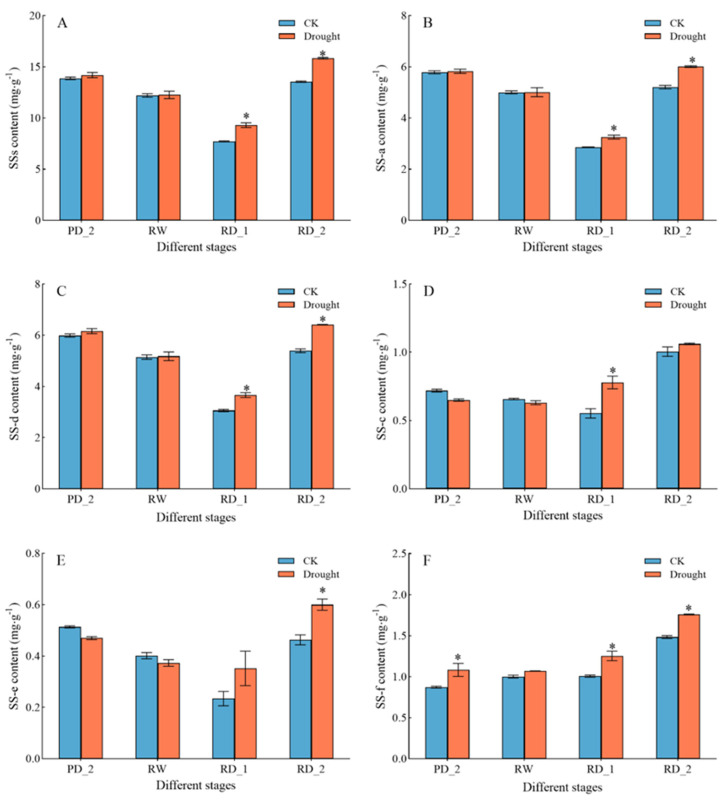
Changes in saikosaponin content under drought stress. (**A**) Changes in saikosaponin content. (**B**) Changes in SS-a content. (**C**) Changes in SS-d content. (**D**) Changes in SS-c content. (**E**) Changes in SS-e content. (**F**) Changes in SS-f content. Data are expressed as means ± SDs (n = 3). * indicates that the drought group and control group significantly differed at the 0.05 level.

Saikosaponin is the main medicinal ingredient in the roots of *B. chinense*. The content of saikosaponin significantly changed under drought stress (Figure 1). Especially at the RD_1 and RD_2 stages, the saikosaponin content increased rapidly under drought stress and peaked at the RD_2 stage. The content of total saikosaponins (SSs) reached 15.85 mg∙g^−1^ (Figure 1A) in the drought group at the RD_2 stage, with a significant increase of 16.94% over the control group (CK) (*p* < 0.05). Five types of saikosaponin also increased in varying degrees. The content of SS-a reached 6.01 mg∙g^−1^ (Figure 1B) in the drought group, with a significant increase of 15.43% over CK (*p* < 0.05). The content of SS-d reached 6.41 mg∙g^−1^ (Figure 1C) in the drought group, with a significant increase of 18.89% over CK (*p* < 0.05). The content of saikosaponin c (SS-c) reached 1.06 mg∙g^−1^ (Figure 1D) in the drought group, with a significant increase of 5.68% over CK (*p* < 0.05). The content of saikosaponin e (SS-e) reached 0.60 mg∙g^−1^ (Figure 1E) in the drought group, with a significant increase of 29.60% over CK (*p* < 0.05). The content of saikosaponin f (SS-f) reached 1.76 mg∙g^−1^ (Figure 1F) in the drought group, with a significant increase of 18.57% over CK (*p* < 0.05). Thus, drought stress had a significant stimulating effect on saikosaponin synthesis, especially at the RD_1 and RD_2 stages. Next, we analyzed gene transcription to understand how drought affects gene expression and ultimately increases saikosaponin content.

### 2.2. Endogenous Hormone Content

**Figure 2:**
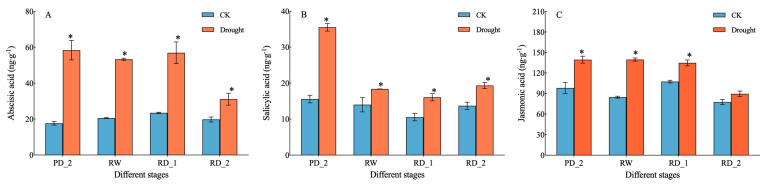
Plant hormone content in roots of *B. chinense* under drought stress. (**A**) Changes in the abscisic acid content. (**B**) Changes in the salicylic acid content. (**C**) Changes in the jasmonic acid content. Data are expressed as means ± SDs (n = 3). * indicates that the drought group and control group significantly differed at the 0.05 level.

Abscisic acid (ABA), salicylic acid (SA), and jasmonic acid (JA) are three plant endogenous hormones closely associated with plant stress resistance. We measured the changes in the content of these three hormones in the roots of *B. chinense* after drought stress (Figure 2). The content of ABA in the drought group was higher than that in CK at different stages (*p* < 0.05). The content of ABA at the pre-drought stage (PD_2), the re-watering stage (RW), and the recurrent drought stage (RD_1) was 58.39, 53.31, and 56.92 ng·g^−1^, which were 3.32, 2.60, and 2.43 times higher than the values of CK, respectively. Although the content of ABA at the RD_2 stage was decreased, it was still 1.57 times that of CK (*p* < 0.05). The content of JA in the drought group was 139.50, 139.64, and 134.77 ng·g^−1^ at the PD_2, RW, and RD_1 stages, which were 1.42, 1.65, and 1.26 times higher than the values of CK, respectively (*p* < 0.05). The content of SA in the drought group was 35.56 ng·g^−1^ at the PD_1 stage, which was 2.28 times that of CK (*p* < 0.05), while the content of SA was slightly higher in the drought group than in CK at the RW and RD_1 and RD_2 stages. When plants are faced with adversity, endogenous hormones respond to environmental changes; ABA and JA had similar response patterns, while SA differed.

### 2.3. Principal Component Analysis (PCA) of Responses to Drought Stress

**Figure 3:**
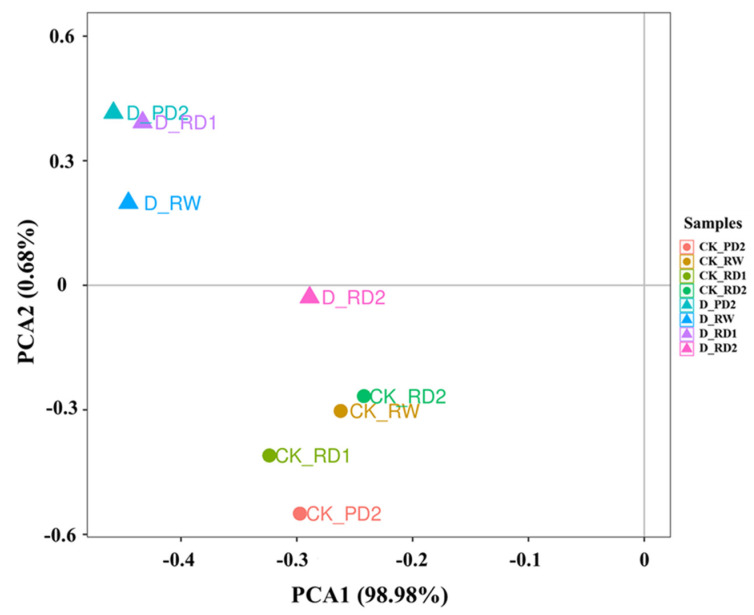
Principal component analysis (PCA) biplot of the first two PCA axes for different *B. chinense* samples under drought stress. Data are expressed as means (n = 3).

Principal component analysis (PCA), a multivariate statistical method, reduces the dimensions of information within a dataset and transforms multiple indicators into a few comprehensive indicators so to better understand complex data more intuitively. The PCA revealed differences between different samples under drought stress (Figure 3); for example, there were significant differences between the drought stress group (D) and the control group (CK). There were no significant differences among PD_2, RW, RD_1, and RD_2 in CK, but the samples PD_2, RW, RD_1, and RD_2 in the drought group separated significantly from CK. This can be explained by the significant changes under drought stress in saikosaponins and the three hormones.

### 2.4. Sequencing Data and Annotation Analysis of B. chinense under Drought Stress

The cDNA library constructed from *B. chinense* roots was sequenced using the Illumina Hiseq 4000 platform (Table 1). A total of 744,141,478 raw reads were generated from 15 samples, and a total of 733,139,232 valid reads were obtained after further removing the sequencing connector and unqualified sequences. The proportion of valid reads was 98.52%, while the data volume of valid reads was 107.81 G. The proportions of Q20 and Q30 in 15 samples were more than 98% and 94%, respectively. The content of GC ranged from 42.05 to 43.95%. In general, the quality of sequencing was high; therefore, the data can be used for subsequent assembly analyses.

A total of 33,379 Unigenes were obtained. The shortest, median, and maximum lengths were 201, 668, and 12,462 bp, respectively. The N50 was 1661 bp. The 200–300 bp Unigenes (9854) were the most common, accounting for 29.52%, followed by the ≥2000 bp Unigenes, (a total of 4451), accounting for 13.33%. The function annotations of Unigenes in National Center for Biotechnology Information (NCBI_NR), Gene Ontology (GO), evolutionary genealogy of genes: Non-supervised Orthologous Groups (eggNOG), Kyoto Encyclopedia of Genes and Genomes (KEGG), Protein families database (Pfam), and Swiss-Prot were carried out using DIAMOND software. All 33,379 Unigenes were annotated in at least one database, and the proportion of annotation was 100% (Table 2). The number of Unigenes annotated to the NR database was the greatest, with 22,320, accounting for 66.87% of all Unigenes (Table S1). In other databases, the numbers of Unigenes annotated, from high to low, were as follows: eggNOG (21,448, 64.26%) (Table S2), GO (19,905, 59.63%) (Table S3), Pfam (17,667, 52.93%) (Table S4), Swiss-Prot (16,554, 49.59%) (Table S5), and KEGG (15,227, 45.62%) (Table S6).

### 2.5. Analysis of DEGs among Different Samples of B. chinense under Drought Stress

**Figure 4:**
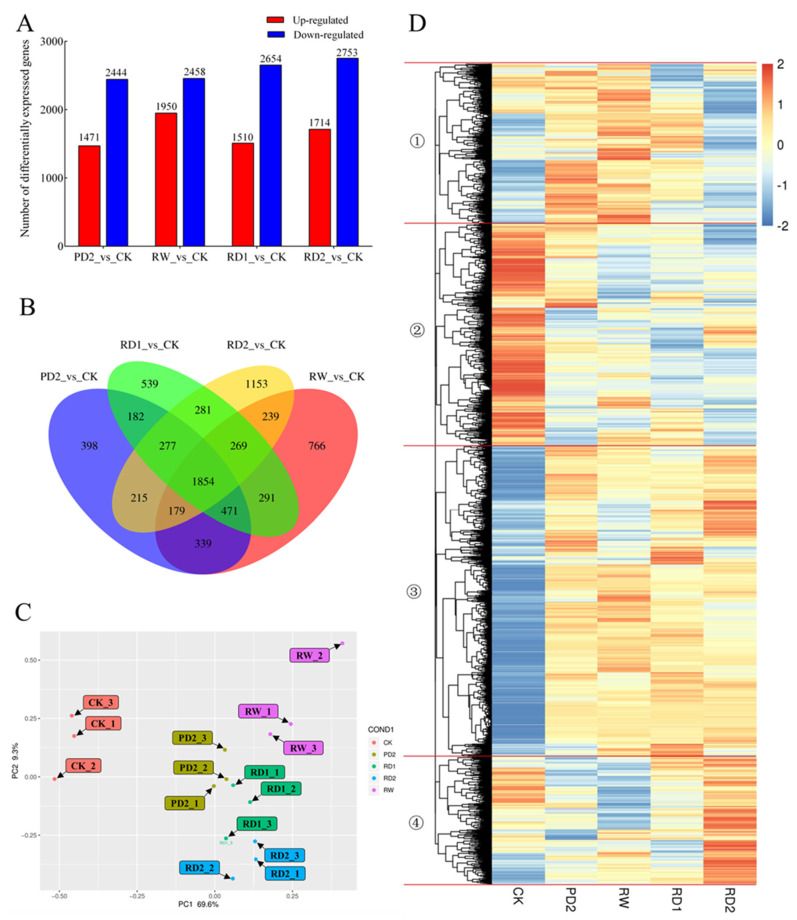
Analysis of differentially expressed genes in *B. chinense* samples under drought stress based on transcriptome sequencing. (**A**) Up-regulation and down-regulation of differentially expressed genes. (**B**) Venn diagrams of differentially expressed genes among different samples. (**C**) PCA of different samples. (**D**) Cluster analysis of differentially expressed genes.

The numbers of differentially expressed genes in the different stages of drought stress (PD_2, RW, PD_1, and PD_2) are shown in Figure 4A. The number of down-regulated genes was higher than that of up-regulated genes in the different stages of drought stress, indicating that drought stress had an inhibitory effect on the gene expression of *B. chinense*. In addition, the RW stage increased the gene expression of *B. chinense* compared with the other three drought stages (PD_2, PD_1, and PD_2). At the transcriptional level, re-watering was beneficial for the recovery of the growth and development of *B. chinense* to a certain extent, and the differential gene expression at the RD_2 stage increased compared with the RD_1 stage, which revealed that *B. chinense* was adaptable to drought stress.

The Venn diagram of the differentially expressed genes in the four comparison groups is shown in Figure 4B. Among them, 1854 genes were differentially expressed in the four comparison groups. The differential expression of these genes may ensure the smooth growth and development of *B. chinense* during drought stress. There were 398, 766, 539, and 1153 differentially expressed genes in PD_2 vs. CK, RW vs. CK, RD_1 vs. CK, and RD_2 vs. CK, respectively. The differential expression of these genes may be the result of the adaptation of *B. chinense* to the different degrees of drought and of time—that is, they reflect the ability of the plant to cope with the different drought stages through the specific expression of different genes.

We also conducted a principal component analysis on the TPM of the Unigenes from different samples (Figure 4C). PC 1 and PC 2 explained 69.6% and 9.3% of the total variance, respectively, accounting for 78.9% of the total variance. There were obvious differences between CK and PD_2, RW, RD_1, and RD_2 in the different stages of drought stress, which indicated that water availability affected gene expression regulation in *B. chinense* and showed the dynamic process of water availability regulating gene expression.

The results of the cluster analysis of differentially expressed genes are shown in Figure 4D. The expression patterns of differentially expressed genes at different stages were divided into four categories: (1) the expression of differentially expressed genes was up-regulated after re-watering, but down-regulated with the return of drought stress; (2) the expression of related genes was down-regulated in the different stages of drought stress; (3) these genes were up-regulated during drought stress; (4) the expression of these genes was significantly up-regulated after the RD_2 stage. The clustering heat map of the differentially expressed genes shows the diversity of the gene expression of *B. chinense* during drought stress, and that various genes are regulated by drought stress, which may affect the various physiological activities of *B. chinense*.

### 2.6. GO and KEGG Functional Annotation and Classification of B. chinense under Drought Stress

The functional classification of GO enrichment under drought stress included translation, structural composition of ribosome, and extracellular region (Figure S1). Drought stress played an important role in regulating plant gene expression and affected the key steps of plant activities. The functional genes related to the translation and structural composition of the ribosome were up-regulated after drought stress (RD_1 and RD_2 stages), which indicated a positive response of *B. chinense* to drought stress.

The KEGG enrichment showed that the metabolic pathways in the PD_2, RD_1, and RD_2 drought stages were ribosome, protein processing in endoplasmic reticulum, plant–pathogen interaction, oxidative phosphorylation, and the mitogen-activated protein kinase (MAPK) signaling pathway cascade (Figure S2). These metabolic pathways are most active under drought stress, and further analysis revealed that the proportions of down-regulated genes decreased gradually from the PD_2 stage to the RD_1 and RD_2 stages, showing that *B. chinense* was adaptable to drought stress.

### 2.7. KEGG Enrichment of MAPK Signaling Pathway—Plant and Plant Hormone Signal Transduction

To further analyze the KEGG enrichment results, we constructed the KEGG pathway of the MAPK signaling pathways plant (map04016), plant hormone signal transduction (map04075), and terpenoid backbone and triterpenoid biosynthesis (map00900 and map00909) to determine the metabolic pathway and the node at which the differentially expressed genes participate.

The MAPK signaling pathway sensed several external signals, as shown in Figure S3. Among them, the phytohormone JA regulates the phosphorylation of MKK3 and MKK6, inhibits MYC2 and VSP2, and ultimately affects root growth and the damage response; ABA regulates multiple genes under drought stress, and affects plant stress tolerance. In addition, other external signals, such as osmotic stress and pathogen attack, are sensed by the MAPK signaling pathway, in which multiple transcription factors such as WRKY33, WRKY229, and ERF1 are involved.

The KEGG pathway of plant hormone signal transduction is shown in Figure S3. ABA regulates the PYR/PYL-PP2C-SNRK2-ABF pathway [25], which ultimately affects stomatal closure and reduces water evaporation under drought stress; JA regulates JAR1-COI1-JAZ-MYC2 and affects ubiquitin-mediated protein degradation and the stress response; SA regulates NPR1-TGA-PR1 to participate in plant disease resistance. In addition to the above three hormones, other hormones involved in plant growth and development, such as auxin, cytokinin, gibberellin, ethylene, and brassinolide, also actively regulate plant hormone signal transduction.

### 2.8. Analysis of Terpenoid Backbone and Triterpenoid Biosynthesis of B. chinense under Drought Stress

**Figure 5:**
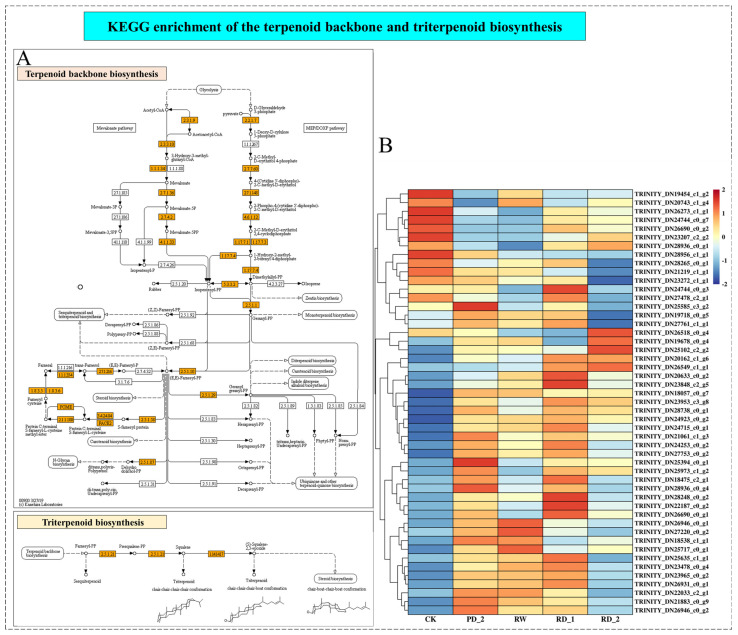
KEGG enrichment of the terpenoid backbone and triterpenoid biosynthesis (**A**) and cluster analysis of the terpenoid backbone and triterpenoid biosynthesis differentially expressed genes (**B**).

For medicinal plants, it is important to analyze the biosynthetic pathway of the active components. Saikosaponin, which belongs to a class of oleanane pentacyclic triterpenoids, is the main active component of *B. chinense*. We constructed the KEGG pathway of the terpenoid backbone and triterpenoid biosynthesis (map00900 and map00909) (Figure 5A). Initially, there were MVA and MEP pathways for the synthesis and transformation of IPP and DMAPP, during which KEGG was enriched to EC: 2.3.1.9 and 15 other enzymes changed. In the second step, IPP and DMAPP were metabolized by farnesyl pyrophosphate synthetase (Z,Z)-farnesyl-PP, (Z,E)-farnesyl-PP, and (E,E)-farnesyl-PP to triterpenoids, during which KEGG was enriched to EC: 2.5.1.10 and 10 other enzymes changed. In the third step, SS and SE were used to catalyze the formation of 2,3-oxysqualene, during which EC: 2.5.1.21 and EC: 1.14.14.17 changed.

Furthermore, we counted the differentially expressed genes enriched in the terpenoid backbone and triterpenoid synthesis pathway and removed the duplicate Unigenes to obtain 49 Unigenes. We constructed the differential gene expression cluster of the terpenoid backbone and triterpenoid synthesis pathway (Figure 5B). Thirty-three differentially expressed genes were significantly up-regulated during drought stress, accounting for 67.4% of all differentially expressed genes. However, only seven differentially expressed genes (14.2%) were significantly down-regulated, and nine differentially expressed genes (18.4%) were down-regulated in the RD_2 stage, indicating that drought stress significantly promoted the synthesis of the terpenoid skeleton and triterpenoids in *B. chinense*.

### 2.9. Analysis of Differentially Expressed Genes Encoding P450 and UGT

**Figure 6:**
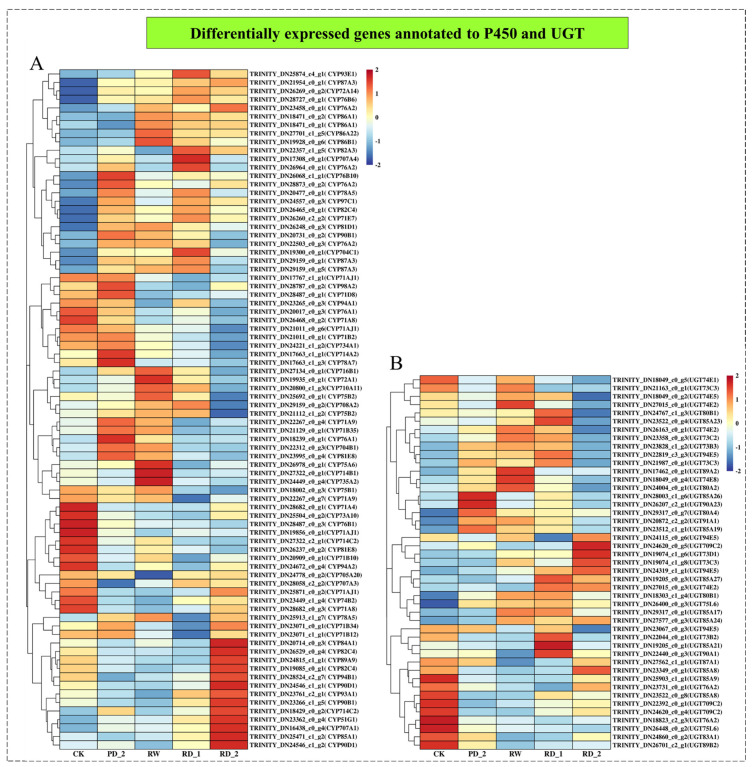
Differentially expressed genes annotated to P450 (**A**) and UGT (**B**) and their cluster analysis.

We used cluster analysis on the annotated P450 and UGT in the differentially expressed genes to screen the possible P450 and UGT involved in saikosaponin synthesis (Figure 6). A total of 80 differentially expressed P450 genes were screened. The genes were divided into three clusters: (1) the expression of P450 was significantly up-regulated in the different stages of drought stress, with 17 Unigenes, accounting for 21.25%; (2) a total of 27 Unigenes were significantly up-regulated at the RD_2 stage, accounting for 33.75%; (3) a total of 36 Unigenes were significantly down-regulated at the RD_1 and RD_2 stages, accounting for 45.00%. In addition, 45 differentially expressed UGT genes were screened, and were roughly clustered into four categories: (1) up-regulation followed by down-regulation; (2) significant down-regulation at the RD_2 stage only; (3) significant up-regulation at the RD_1 and RD_2 stage; (4) significant down-regulation at the different stages.

According to the expression patterns of P450 and UGT, we screened the P450 and UGT, which were consistent with the change in saikosaponin content. One P450 gene (TRINITY_DN23071_c0_g1) was negatively correlated with saikosaponin content (the correlation coefficient was −0.970, *p* < 0.05). TRINITY_DN23071_c0_G1 was annotated as CYP71B34, and GO annotation showed that it had oxygenase activity and participated in the synthesis of secondary metabolites. Correlation analysis also revealed that two UGTs were related to the change in saikosaponin content. The correlation between TRINITY_DN24767_c1_g3 and saikosaponin content was 0.959 (*p* < 0.05); it was annotated as UGT80B1, and GO annotation showed that it had glucosyltransferase activity. The correlation between TRINITY_DN23522_c0_g4 and saikosaponin content was 0.996 (*p* < 0.01); it was annotated as UGT85A23, and GO annotation showed that it also had glucosyltransferase activity. In conclusion, one P450 (CYP71B34) and two UGTs (UGT80B1 and UGT85A23) may be involved in the saikosaponin synthesis pathway; further analysis is needed to verify their specific functions in saikosaponin synthesis.

### 2.10. Analysis of Differentially Expressed Genes of Transcription Factors

**Figure 7:**
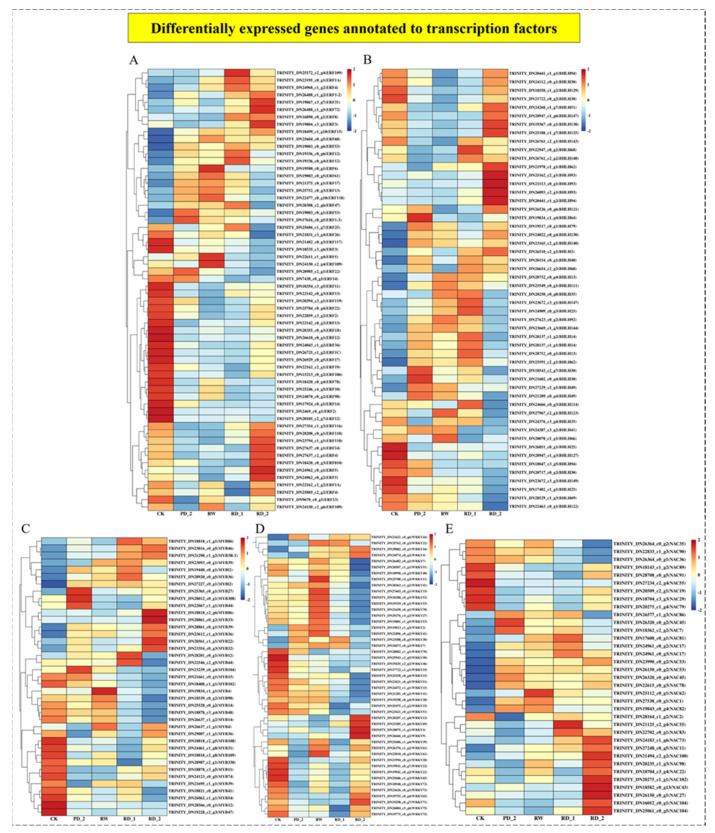
Differentially expressed genes annotated to ERF (**A**), BHLB (**B**), MYB (**C**), WRKY (**D**), and NAC (**E**) and their cluster analysis.

Transcription factors can initiate and regulate gene expression by recognizing and binding cis-acting elements in the gene promoter region [26]. To screen for transcription factors that possibly participate in the saikosaponin synthesis pathway, we clustered the transcription factor families ethylene-responsive factor (ERF), basic/helix-loop-helix (bHLH), MYB transcription factor (MYB), WRKY transcription factor (WRKY), and NAC transcription factor (NAC) in the differentially expressed genes (Figure 7) and annotated a total of 233 genes, including 60 ERF, 52 bHLH, 39 MYB, 47 WRKY, and 35 NAC. In different drought stages, various transcription factors of *Bupleurum chinense* rapidly responded to adverse environmental changes, thereby enabling plants to adapt to adversity. Various transcription factors were significantly up-regulated; ERF had the largest number of up-regulated genes in the RD_2 stage, with 28 ERF genes up-regulated (*p* < 0.05), accounting for 46.7%. bHLH had the largest number of up-regulated genes in the RD_1 stage, with 28 bHLH genes up-regulated (*p* < 0.05), accounting for 53.8%. MYB had the largest number of up-regulated genes in the PD_2 stage, RW stage, and RD_2 stage, with 17 MYB genes up-regulated (*p* < 0.05), accounting for 43.6%. WRKY had the largest number of up-regulated genes in the RW stage, with 23 WRKY genes up-regulated (*p* < 0.05), accounting for 48.9%. NAC had the largest number of up-regulated genes in the RD_2 stage, with 24 NAC genes up-regulated (*p* < 0.05), accounting for 68.6%. Different transcription factor families had different expression patterns in different drought stress stages.

### 2.11. Expression of Key Genes Encoding Saikosaponin Biosynthesis Enzymes in Response to Drought Stress

**Figure 8:**
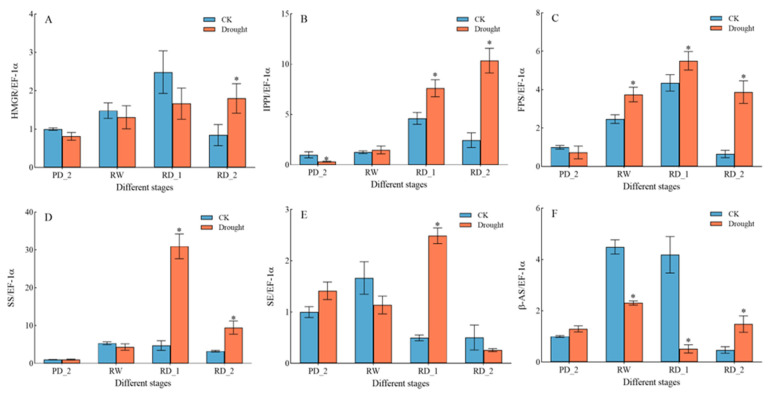
Effects of drought stress on the expression of the key saikosaponin biosynthesis enzyme genes. Expression levels of (**A**) *HMGR* gene, (**B**) *IPPI* gene, (**C**) *FPS* gene, (**D**) *SS* gene, (**E**) *SE* gene, and (**F**) *β-AS* gene. Data are expressed as means ± SDs (n = 3). * indicates that the drought group and control group significantly differed at the 0.05 level.

We used qPCR analysis to confirm the accuracy of the transcriptome data and better understand the expression of saikosaponin synthesis-related genes. The results of the qPCR analysis of the *HMGR*, *IPPI*, *FPS*, *SS*, *SE*, and *β-AS* genes under drought stress are shown in Figure 8. The expression of different genes was not consistent under drought stress. The *HMGR* gene was significantly up-regulated only in the RD_2 stage, with an up-regulation multiple of 2.13. The *IPPI* gene was significantly up-regulated in the RD_1 and RD_2 stages, with up-regulation multiples of 1.65 and 4.22, respectively. The *FPS* gene was significantly up-regulated in the RW, the RD_1, and the RD_2 stages, with up-regulation multiples of 1.52, 1.26, and 5.97 respectively. The *SS* gene was significantly up-regulated in the RD_1 and the RD_2 stages, with up-regulation multiples of 6.52 and 2.92, respectively. The *SE* gene was significantly up-regulated only in the RD_1 stage, with an up-regulation multiple of 5.01. The *β-AS* gene was significantly up-regulated only in the RD_2 stage, with an up-regulation multiple of 3.14.

### 2.12. Screening of Transcription Factors Involved in Saikosaponin Synthesis Regulation

To screen for the transcription factors possibly involved in the regulation of saikosaponin synthesis, we analyzed the correlations between the selected transcription factors in the transcriptome database and the key genes encoding enzymes for the synthesis of saikosaponin. ERF109 (TRINITY_DN24150_c2_g4) was positively correlated with the *FPS* gene (*p* < 0.01). WRKY40 (TRINITY_DN18990_c4_g3), ERF1–3 (TRINITY_DN17616_c0_g5), and NAC53 (TRINITY_DN26150_c0_g2) were positively correlated with the *FPS* gene (*p* < 0.05). ERF1–2 (TRINITY_DN26488_c1_g2), MYB48 (TRINITY_DN18878_c3_g4), and bHLH144 (TRINITY_DN23669_c1_g3) were positively correlated with the *SE* gene (*p* < 0.05). bHLH14 (TRINITY_DN28137_c1_g2) was positively correlated with the *β-AS* gene (*p* < 0.05). These eight transcription factors screened rapidly responded to drought stress and regulated the expression of related key genes encoding enzymes, which may be important transcription factors involved in the regulation of saikosaponin biosynthesis.

### 2.13. Preliminary Construction of the Saikosaponin Synthesis Regulation Network

**Figure 9:**
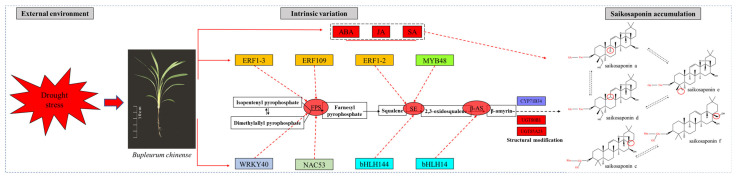
The saikosaponin synthesis regulation network under drought stress.

Based on the above analysis, we preliminarily constructed the saikosaponin synthesis regulation network by combining the results of the correlation analysis of the transcriptomic high-throughput data, the qPCR high-sensitivity gene expression detection, the hormone content analysis via UHPLC-ESI-MS/MS, and the saikosaponin change analysis via HPLC. Bioinformatic analysis was performed using the OmicStudio tools (https://www.omicstudio.cn/tool). The saikosaponin synthesis regulation network is illustrated in Figure 9. Under the stimulation of drought stress, WRKY40, ERF1–2, ERF1–3, ERF109, MYB48, bHLH14, bHLH 144, and NAC53 rapidly responded, regulated the expression of the *FPS*, *SE*, and *β-AS* genes, and promoted the synthesis of saikosaponins. These results provide new insights into the synthesis of saikosaponins under drought stress.

## 3. Discussion

Plants produce secondary metabolites to adapt to the changes of the external environment [27]. At the same time, secondary metabolites are beneficial for drug development [28]. Understanding the process of adaptation of *B. chinense* to the external environment (drought stress) and revealing the biosynthesis of saikosaponins in vivo are particularly important for the production of stable, reliable, and effective traditional Chinese medicinal materials [29]. In the present study, we used RNA-seq technology for the first time to analyze how drought stress stimulated the terpene main chain and triterpene biosynthesis pathway in *B. chinense* to promote the synthesis of saikosaponins and analyzed the relationship between the transcriptional information and the changes in endogenous hormones during drought stress. Several CYP450 and UGT genes possibly involved in saikosaponin synthesis were screened; the expression pattern of the key genes encoding enzymes for the synthesis of saikosaponins was analyzed using qPCR. The transcription factors ERF, BHLH, MYB, WRKY, and NAC, which may regulate the key genes of saikosaponin synthesis, were screened, and the regulatory network of saikosaponin synthesis was constructed. Our results provide insights into the molecular mechanism underlying the formation of saikosaponins in *B. chinense* under drought stress, which has great importance for optimizing the water supply strategy and cultivation technology for *B. chinense.*

Most importantly, the accumulation of saikosaponins in *B. chinense* was changed significantly under drought stress [30]. Moreover, under the stimulation of drought stress, the accumulation of saikosaponins showed an obvious short-term stress effect. The drastic change in soil water after re-watering following drought stress (the RW, RD_1, and RD_2 stages) had a significant impact on saikosaponins. At the RW stage, the soil water content returned to the normal level. When drought recurred (the RD_1 stage), the content of saikosaponins in the roots of *B. chinense* decreased, possibly because *B. chinense* was watered in the previous stage. The synthesis of saikosaponins was temporarily inhibited, and physiological activities were directed to the accumulation of dry matter from the growth of roots. With the continuing development of drought stress (the RD_2 stage), re-stimulation by the drought stress signal effectively promoted the synthesis of saikosaponins. The content of SS-a and SS-d increased by 84.60% and 75.13%, respectively, from the RD_1 stage to the RD_2 stage, indicating that the synthesis of saikosaponins, a secondary metabolite in the roots of *B. chinense*, responded rapidly to the change in soil water content. The changeable external environment (soil water content) had an obvious short-term stress effect on the synthesis of saikosaponins. Similar results were also reflected in the drought stress response of *Scutellaria baicalensis* Georgi. Cheng et al. proposed that an appropriate degree of drought stress may promote the accumulation of baicalin by stimulating the expression and activities of the key enzymes involved in baicalin biosynthesis [31]. The best time for observing the short-term stress effect was when *B. chinense* plants re-encountered drought stress following re-watering, indicating that *B. chinense* should be harvested after precipitation and when the soil is dry again. At this time, the synthesis of saikosaponins was promoted by the stimulation by repeated drought stress; it is a critical time for determining the yield and quality of the *B. chinense* harvest.

The synthesis and accumulation of secondary metabolites in medicinal plants are affected by time (different development stages), space (different tissue parts), and environmental conditions (different ecological factors) [32]. We studied the adaptation of *B. chinense* to drought stress, and the expression levels of key genes encoding enzymes for the biosynthesis of saikosaponins, by constructing transcriptome libraries of *B. chinense* under different drought stresses, to reveal the process of adaptation of *B. chinense* to drought stress, and the pathway and mechanism of the regulation of secondary metabolite biosynthesis. The expression patterns of differential genes at different stages can be divided into four categories. The gene expression of *B. chinense* varied while adapting to drought stress. Many genes are regulated by drought stress, which may affect the various life activities of *B. chinense*. Moreover, the results of GO and KEGG enrichment revealed the overall adaptability of different genes’ expression to drought stress. The number of up-regulated genes among related genes increased significantly at the RD_2 and RD_1 stages, especially compared with the PD_2 stage, indicating that the plants had an adaptability to drought stress. A more important discovery was in the plant hormone signal transduction pathway: ABA regulates the PYR/PYL-PP2C-SnRK2-ABF pathway and ultimately affects plant stomatal closure; JA regulates JAR1-COI1-JAZ-MYC2 and affects ubiquitin-mediated protein degradation and the stress response; SA regulates NPR1-TGA-PR1 and participates in the process of plant disease resistance. These endogenous hormones responded to environmental changes by increasing the content of ABA, JA, and SA rapidly during drought stress; consequently, through the mechanisms in the plant MAPK signaling pathway and plant hormone signal transduction, the three plant hormones may jointly affect the adaptation of *B. chinense* to drought stress [33,34].

For medicinal plants, the analysis of the biosynthetic pathways of their pharmacodynamic components is important [35]. The main pharmacodynamic component of *B. chinense* is saikosaponin, a class of oleanolane pentacyclic triterpenoids [36]. Through the analysis of the terpene skeleton and triterpene synthesis pathway, we screened 49 Unigenes, of which 33 differential genes were significantly up-regulated during drought stress, accounting for approximately 67.4% of all differential genes. Although drought stress inhibited the expression of most genes, it significantly stimulated the up-regulation of the expression of genes related to the terpene skeleton and triterpene synthesis pathway. The expression of related genes was up-regulated and saikosaponins were finally synthesized, which was consistent with the increase in saikosaponin content. Moreover, according to the correlation analysis of the expression patterns of P450 and UGT, we identified one P450 (TRINITY_DN23071_c0_g1, annotated as CYP71B34 gene) that had a correlation of −0.970 (*p* < 0.05) with the change in saikosaponin content, and two UGTs were related to the change in saikosaponin content, of which TRINITY_DN24767_c1_g3 had a correlation of 0.959 (*p* < 0.05), annotated as UGT80B1, and TRINITY_DN23522_c0_g4 had a correlation of 0.996 (*p* < 0.01) with the change in saikosaponin content, annotated as UGT85A23. Further GO annotation analysis of the above three genes showed that CYP71B34 had oxygenase activity [37] and UGT80B1 and UGT85A23 had glucosyltransferase activity [38,39]. We speculate that the above genes may participate in the structural modification of saikosaponins, affect the structural diversity of saikosaponins, and then produce saikosaponins with various structures. Further, we verified the expression changes of six key genes encoding enzymes for the synthesis of saikosaponins under drought stress, and screened the transcription factors (WRKY40, ERF1-2, ERF1-3, ERF109, MYB48, bHLH14, bHLH 144, and NAC53) that may be involved in regulating the expression of key genes encoding enzymes according to the transcription of transcription factor families. To summarize, this study combined transcriptome analysis, qPCR, l UHPLC-ESI-MS/MS, HPLC, and other techniques to reveal the molecular network of saikosaponin synthesis in *B. chinense* under drought stress. Understanding the regulatory network of saikosaponin synthesis, involving external drought stimulation, transcription factors, and key enzyme genes, will provide deeper insights into saikosaponin biosynthesis in *B. chinense*.

## 4. Conclusions

Herein, we investigated how drought stress stimulates the transcriptional changes of *B. chinense* and increases the synthesis of saikosaponins. The accumulation of saikosaponins showed a short-term effect under the stimulation of drought stress, especially from the RD_1 stage to the RD_2 stage, when drought stress stimulated the terpenoid backbone and triterpenoid biosynthesis pathway to promote the synthesis of saikosaponins. At the same time, the drought stress stimulated a rapid increase in the content of three plant hormones. Moreover, we identified that one P450 and two UGTs may be involved in the synthesis of saikosaponins, and eight transcription factors (WRKY40, ERF1–2, ERF1–3, ERF109, MYB48, bHLH14, bHLH 144, and NAC53) are possibly involved in the regulation of key enzyme genes’ expression in saikosaponin synthesis. The regulation network for the synthesis of saikosaponins under drought stress was constructed based on transcription factors, the expression of key enzyme-encoding genes, and the synthesis of saikosaponins. Our results provide insights into improving the cultivation of *B. chinense*. When harvesting *B. chinense*, the reduction of saikosaponin content by excessive rain should be avoided; the best time to harvest is after a period of drought stress. This study provides a practical means to ensure the quality (safety and effectiveness) of *B. chinense*, and promotes the modernization of the China Agriculture Research System (CARS).

## 5. Materials and Methods

### 5.1. Experimental Treatment

The experimental site was located at the Agricultural Base of Jilin Agricultural University (D08 Medicinal Plant Cultivation and Physiological and Ecological Control Practice Teaching Station). Dried and mature seeds of *B. chinense* were collected from the Medicinal Botanical Garden of the university, planted in pots, and maintained by conventional field management.

The drought stress experiment began after *B. chinense* had grown enough biomass (three months after germination). The control group (CK) were maintained under normal water supply (1500 mL each time, according to our previous experiment’s plan). The drought group were maintained with half the water supply of the control group (drought; 750 mL each time) during the pre-drought stage (PD). After the pre-drought stage, water was applied once (re-watering stage, RW) and the plants were then subjected to drought stress again (recurrent drought stage, RD). A more detailed experimental design was previously reported [30]. Samples were collected from 7:00 a.m. to 8:00 a.m. the day before the next water supply was provided. Ten plants of each treatment were collected for one biological repeat, and each treatment was biologically repeated 3 times. We selected PD_2, RW, RD_1, and RD_2 samples described therein for HPLC, UHPLC-ESI-MS/MS, and transcriptome analyses.

### 5.2. Determination of Saikosaponin Content in Roots

We used HPLC as the elution procedure to determine saikosaponin content, as previously described [40]. Reference standards saikosaponin a (mass fraction ≥ 98%, batch number B20146), saikosaponin c (mass fraction ≥ 98%, batch number B20149), saikosaponin d (mass fraction ≥ 98%, batch number b20150), saikosaponin e (mass fraction ≥ 98%, batch number B24458), saikosaponin f (mass fraction ≥ 98%, batch number B20151) were purchased from Shanghai Yuanye Bio-Technology Co., Ltd. The saikosaponins determined included saikosaponin a (SS-a), saikosaponin d (SS-d), saikosaponin c (SS-c), saikosaponin e (SS-e), and saikosaponin f (SS-f), and their total content was used to calculate the content of saikosaponins (SSs).

### 5.3. Determination of Endogenous Hormone Content

Abscisic acid (ABA), salicylic acid (SA), and jasmonic acid (JA) in *B. chinense* roots were determined using UHPLC-ESI-MS/MS. Extraction of endogenous hormones: 0.1 g of *B. chinense* root was preserved at −80 °C and ground to a fine powder in liquid nitrogen, transferred into a 1.5 mL centrifuge tube filled with 1 mL 80% methanol (containing 0.3 ng isotope internal standard), placed in a mixer for 1 h (4 °C), and centrifuged at 14,000× *g* for 10 min (4 °C). The supernatant was transferred to a precooled 2 mL centrifuge tube; the remaining solid was extracted with 1 mL 80% methanol, mixed in a mixer for 1 h (4 °C), and centrifugated at 10 min (4 °C). After centrifugation, the supernatant was mixed twice; then, it was blown dry with a nitrogen blower, 30% methyl alcohol was added, and it was mixed for 1 h (4 °C) and centrifuged at 14,000× *g* for 10 min (4 °C); the supernatant was transferred to a sample tube, and the ABA, SA, and JA content was detected using UHPLC-ESI-MS/MS.

The endogenous hormones were detected using an ultra-performance liquid chromatography Acquity UPLC BEH C18 column (Waters Corp, Milford, MA, USA; 2.1 mm × 150 mm, 1.7 μm); mobile phase: methanol (A)–water (B), gradient elution in negative ion mode (0~1 min, 15% A; 1–8 min, 15% A → 100% A; 8–9.5 min, 100% A; 9.5–12 min, 100% A → 15% A); column temperature: 40 °C; injection volume: 20 μL; flow rate: 0.3 mL·min^−1^. Detection mode: MRM; ionization mode: electrospray ionization (ESI); ionization voltage (IS): 4.5 kV; ion source temperature: 500 °C; air curtain pressure: 40 psi; spray pressure: 40 psi; auxiliary heating pressure: 40 psi; mass spectrometry acquisition parameters as shown in Table 3. Deuterium-labeled [^2^H_6_] (+)-2-*cis*-4-*trans*-abscisic acid was used as an internal standard. The content of three endogenous hormones in *B. chinense* roots was calculated according to Formula (1):

(1) $$\mathrm{Hormone}\;\mathrm{content}\;(\mathrm{ng}\;\times \;g^{-1})\;=\;\frac{\mathrm{Area}\;\mathrm{of}\;\mathrm{hormone}\;\mathrm{peak}\;\mathrm{in}\;\mathrm{sample}\;\times \;\mathrm{Mass}\;\mathrm{of}\;\mathrm{isotope}\;\mathrm{internal}\;\mathrm{standard}\;}{\mathrm{Area}\;\mathrm{of}\;\mathrm{isotopic}\;\mathrm{internal}\;\mathrm{standard}\;\times \;\mathrm{Mass}\;\mathrm{of}\;\mathrm{samples}}$$

### 5.4. Identification and Functional Annotation of Differentially Expressed Genes

#### 5.4.1. Total RNA Extraction and Sequencing

The total RNA of *B. chinense* roots was extracted using Trizol reagent (Invitrogen, Carlsbad, CA, USA), following the manufacturer’s procedure. The total RNA quantity and purity were analyzed using Bioanalyzer 2100 and RNA 1000 Nano LabChip Kits (Agilent, CA, USA) with RIN number > 7.0. Poly(A) RNA was purified from total RNA (5 μg) using poly-T oligo-attached magnetic beads, using two rounds of purification. Following purification, the mRNA was fragmented into small pieces using divalent cations under elevated temperature. Then, the cleaved RNA fragments were reverse-transcribed to create the final cDNA library, in accordance with the protocol for the mRNASeq sample preparation kit (Illumina, San Diego, CA, USA); the average insert size for the paired-end libraries was 300 bp (±50 bp). Paired-end sequencing was performed on an Illumina Hiseq 4000 (Illumina, Inc., San Diego, CA, USA) following the vendor’s recommended protocol by Lc-Bio Technologies (Hangzhou, China) Co., Ltd.

#### 5.4.2. De Novo Assembly, Unigene Annotation, and Functional Classification

Firstly, Cutadapt [41] and perl scripts developed in-house were used to remove the reads that contained adaptor contamination, low-quality bases, and undetermined bases. Sequence quality, including the Q20, Q30, and GC content of the clean data, was verified using FastQC. All downstream analyses were based on clean data of high quality. De novo assembly of the transcriptome was performed with Trinity 2.4.0 [42]. Trinity groups transcripts into clusters based on shared sequence content. Such a transcript cluster is very loosely referred to as a ‘gene’. The longest transcript in the cluster was chosen as the ‘gene’ sequence (aka Unigene). All assembled Unigenes were aligned against the non-redundant (Nr) protein database, Gene Ontology (GO), SwissProt, Kyoto Encyclopedia of Genes and Genomes (KEGG), and eggNOG databases using DIAMOND [43], with a threshold of E value < 0.00001.

#### 5.4.3. Differentially Expressed Gene (DEG) Analysis

Salmon [44] was used to estimate expression levels for Unigenes by calculating transcripts per million (TPM) [45]. The differentially expressed Unigenes were selected with log_2_(fold change) > 1 or log_2_(fold change) < −1 and with statistical significance (*p* value < 0.05) using the R package edgeR [46]. Next, GO and KEGG enrichment analyses were used on the differentially expressed Unigenes using in-house perl scripts.

### 5.5. Determination of Key Genes in the Saikosaponin Synthesis Pathway

To further clarify the expression of key enzyme genes in the saikosaponin synthesis pathway, we used quantitative real-time PCR (qPCR) to detect the key enzyme genes previously reported. The selected key enzyme genes included 3-hydroxy-3-methylglutaryl-CoA reductase (*HMGR*), isopentenyl diphosphate isomerase (*IPPI*), farnesyl pyrophosphate synthase (*FPS*), squalene synthase (*SS*), squalene epoxidase (*SE*), and β-amyrin synthase (*β-AS*) [11]. The elongation factor 1α (EF-1α) housekeeping gene was used as a reference gene to evaluate the relative gene expression levels. A qRT-PCR analysis was completed using an Mx 3000P instrument (Agilent, Santa Clara, CA, USA). The primers for each gene reaction procedure have been previously reported [17]. Each 20 μL PCR contained 10 μL of 2 × SYBR Premix Ex Taq, and the optimum amounts of primers and cDNA were 0.8 μL and 1.0 μL, respectively. The reaction sequence was as follows: 30 s at 94 °C for pre-denaturation, 5 s at 95 °C for denaturation, 30 s at 55 °C for annealing, and 20 s at 72 °C for extension. This thermal cycle was repeated 40 times. Gene expression was calculated using the 2^−ΔΔCt^ method.

### 5.6. Statistical Analysis

Single-factor analysis of variance and Pearson correlation analysis were performed using SPSS 19.0. Excel 2016 was used to arrange the raw data. GraphPad Prism 8.0 was used to graph the data.

## Figures and Tables

**Table 1:** RNA-seq data for *B. chinense* root samples.

Sample	Raw Reads	Raw Bases	Valid Reads	Valid Bases	Valid (%)	Q20%	Q30%	GC%
CK_1	54,026,434	7.62 G	53,451,132	7.45 G	98.94	98.26	94.77	43.95
CK_2	56,586,800	7.98 G	55,938,322	7.82 G	98.85	98.46	94.99	43.92
CK_3	53,820,460	7.59 G	53,229,136	7.44 G	98.90	98.44	94.91	43.20
PD2_1	41,709,950	6.26 G	39,579,492	5.90 G	94.89	98.72	95.48	42.87
PD2_2	42,729,440	6.41 G	42,223,028	6.31 G	98.81	98.91	95.82	42.47
PD2_3	44,270,858	6.64 G	43,101,032	6.43 G	97.36	98.84	95.73	42.42
RW_1	43,486,690	6.52 G	42,148,790	6.29 G	96.92	98.81	95.63	42.27
RW_2	49,878,582	7.48 G	49,477,892	7.37 G	99.20	98.81	95.72	42.05
RW_3	50,165,512	7.52 G	49,848,972	7.44 G	99.37	98.90	95.87	42.34
RD1_1	45,543,552	6.83 G	45,263,368	6.75 G	99.38	98.90	95.91	42.37
RD1_2	46,609,874	6.99 G	46,239,616	6.89 G	99.21	98.83	95.77	42.38
RD1_3	52,905,820	7.94 G	51,958,506	7.74 G	98.21	98.73	95.47	42.62
RD2_1	49,930,204	7.49 G	49,394,108	7.37 G	98.93	98.84	95.68	42.30
RD2_2	57,051,090	8.56 G	56,531,180	8.44 G	99.09	98.83	95.67	42.43
RD2_3	55,426,212	8.31 G	54,754,658	8.17 G	98.79	98.80	95.64	42.47

**Table 2:** Annotation results.

Databases	Number of Unigenes Annotated	Ratio (%)
NR	22,320	66.87
GO	19,905	59.63
eggNOG	21,448	64.26
KEGG	15,227	45.62
Pfam	17,667	52.93
Swiss-Prot	16,554	49.59
All	33,379	100.00

**Table 3:** Retention time, characteristic ion analysis via hormone UHPLC-ESI-MS/MS.

Hormone	Parent Ion (m∙s^−1^)	Daughter Ion (m∙s^−1^)	Retention Time (min)
ABA	262.9	153.1	6.09
SA	137.0	93.0	5.81
JA	209.0	58.9	6.50

## Data Availability

The datasets presented here can be found in online repositories (National Center for Biotechnology Information), under accession number PRJNA795661 (https://www.ncbi.nlm.nih.gov/bioproject/PRJNA795661, accessed on 8 January 2022).

## Supplementary Materials

The following supporting information can be downloaded at: https://www.mdpi.com/article/10.3390/molecules27175470/s1.
