# Supplementary material for: Drought Stress Stimulates the Terpenoid Backbone and Triterpenoid Biosynthesis Pathway to Promote the Synthesis of Saikosaponin in Bupleurum chinense DC. Roots
Source: Molecules. 2022 Aug 25;27(17):5470. doi: 10.3390/molecules27175470 (PMC9457724; doi:10.3390/molecules27175470)
Supplement: Supplementary file 1 [file molecules-27-05470-s001.zip › Supplementary Material. Figure S1.pdf]

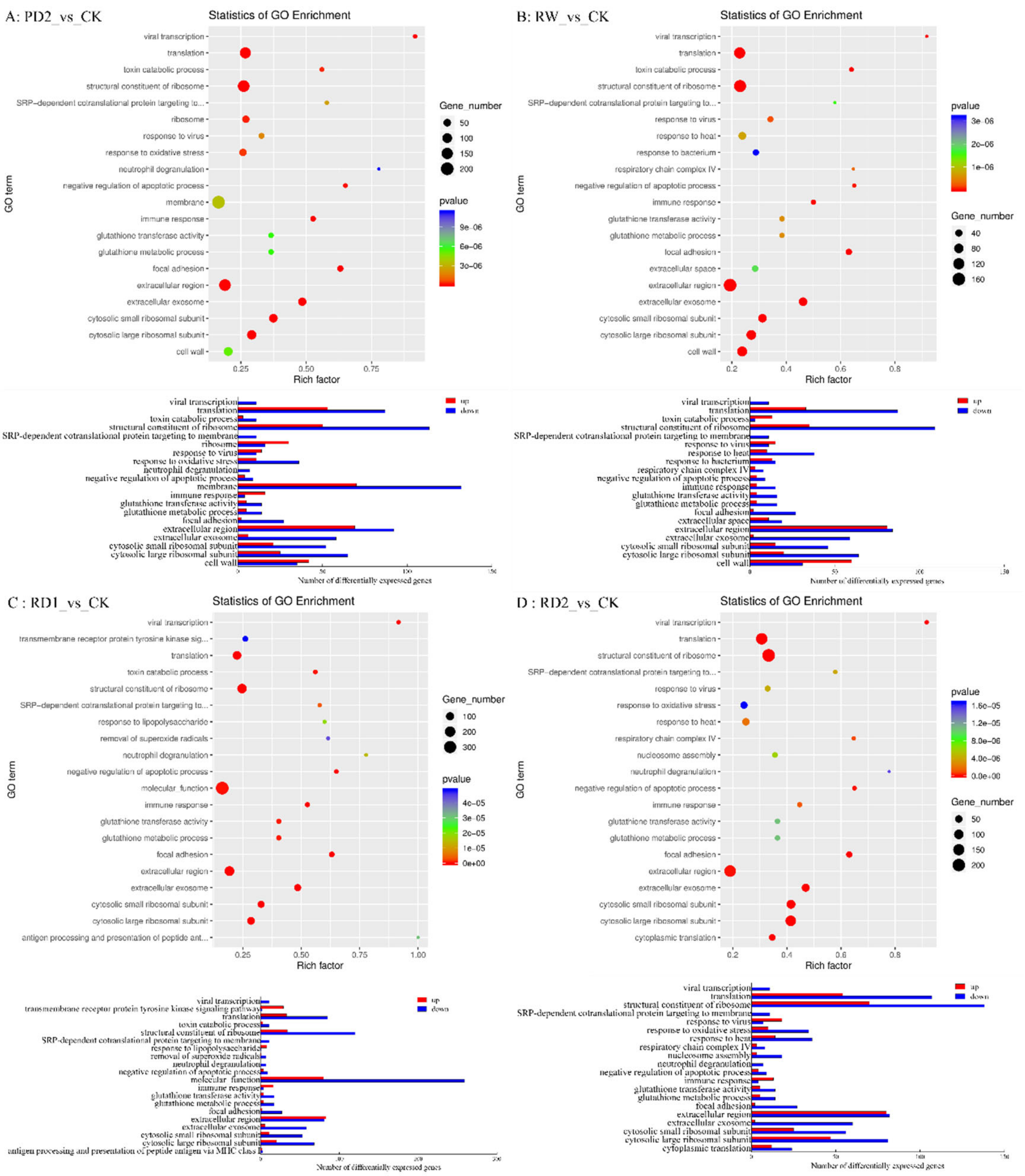

Figure S1. GO enrichment analysis of differentially expressed genes (GO enrichment scatterplot and GO classification of up- and down-regulated differentially expressed genes). (A) PD2 vs CK. (B) RW vs CK. (C) RD1 vs CK. (D) RD2 vs CK.
