# Supplementary material for: Drought Stress Stimulates the Terpenoid Backbone and Triterpenoid Biosynthesis Pathway to Promote the Synthesis of Saikosaponin in Bupleurum chinense DC. Roots
Source: Molecules. 2022 Aug 25;27(17):5470. doi: 10.3390/molecules27175470 (PMC9457724; doi:10.3390/molecules27175470)
Supplement: Supplementary file 1 [file molecules-27-05470-s001.zip › Supplementary Material. Figure S3.pdf]

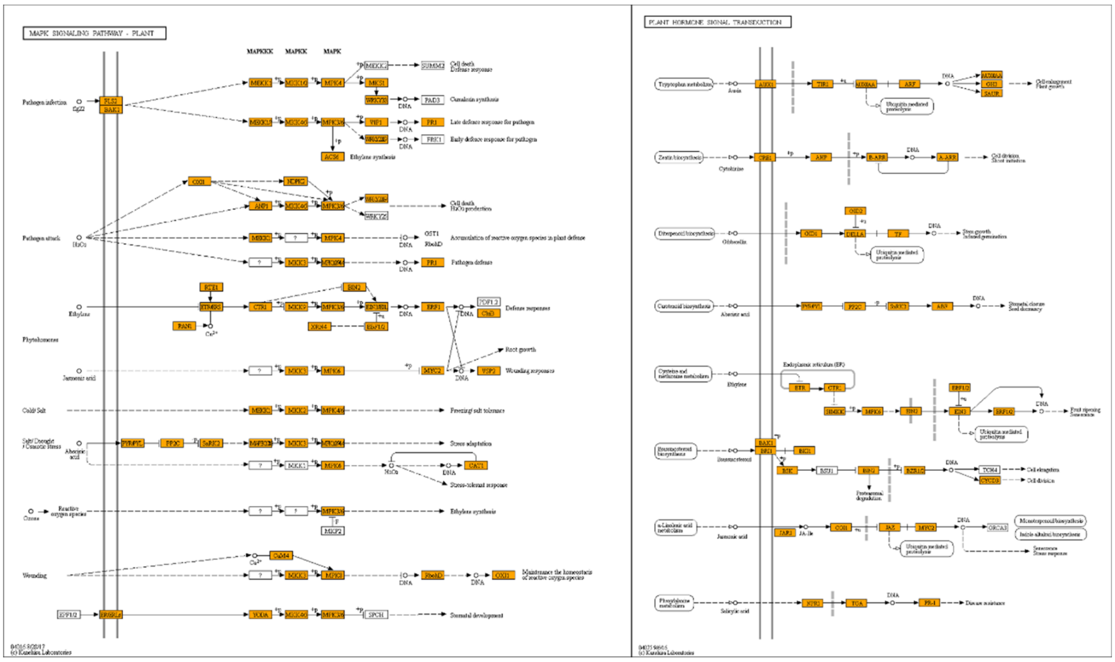

Figure S3. KEGG enrichment of the MAPK signaling pathway-plant and plant hormone signal transduction.
